# Snacking for a reason: detangling effects of socio-economic position and stress on snacking behaviour

**DOI:** 10.1186/s12889-022-14384-2

**Published:** 2022-11-02

**Authors:** Marleen Gillebaart, Caroline Schlinkert, Maartje P. Poelman, Jeroen S. Benjamins, Denise T.D. De Ridder

**Affiliations:** 1 Social, Health and Organisational Psychology, Utrecht University, 3508TC Utrecht, PO Box 80140, The Netherlands; 2 Chair group Consumption and Healthy Lifestyles, Wageningen University & Research, 6700 EW Wageningen, P.O. Box 8130, The Netherlands

**Keywords:** Snacking behaviour, Socio-economic position, Life stress, COVID-19 related worry, Reasons for snacking

## Abstract

**Background:**

As snacking can be considered a cornerstone of an unhealthy diet, investigating psychological drivers of snacking behaviour is urgent, and therefore the purpose of this study. Socio-economic position (SEP) and stress are known to affect many behaviours and outcomes, and were therefore focal points in the study.

**Methods:**

In a cross-sectional survey study, we examined whether Socio-economic position (SEP) would amplify associations between heightened stress levels and self-reported negative-affect related reasons for snacking. Next, we investigated whether Socio-economic position (SEP) predicted frequency of snacking behaviour, and how stress and other reasons for snacking could explain this association. Outcome measures were reasons people indicated for snacking, and frequency of snacking behaviour.

**Results:**

Analyses revealed that people seem to find more reasons to snack when they are stressed, and that this association was more pronounced for people with a high compared to low socio-economic position. Furthermore, a higher socio-economic position was associated with a higher frequency of snacking, and both snacking to reward oneself and snacking because of the opportunity to do so remained significant mediators.

**Conclusion:**

Whereas low socio-economic position was associated with higher stress levels, this did not translate into increased snacking. Contrarily, those with higher socio-economic position could be more prone to using ‘reasons to snack’, which may result in justification of unhealthy snacking behaviour.

**Supplementary Information:**

The online version contains supplementary material available at 10.1186/s12889-022-14384-2.

The contribution of snacking to our energy intake increased substantially over the past decades, to a current total of 25–35% of daily energy intake that may come from snacking [1]. In turn, increased energy intake significantly contributed to increased overweight and obesity prevalence globally [2], illustrating the urgency of studying why and how people snack. Socioeconomic position (SEP; the social status of an individual or group, often assessed as (a combination of) education, income, and occupation; [3]) has a pervasive impact on diet, health, and wellbeing [4–6]. Prior studies show potential links between SEP and snacking specifically [7, 8] and that psychological factors associated with eating behaviours, like stress, are more pronounced in lower than in higher SEP groups. Stress can be described as what occurs when environmental demands exceed one’s perception of one’s ability to cope with those demands [9] and is often accompanied by feelings of psychological and/or physical discomfort. Stress has also been linked to changes in snacking behaviour [10, 11]. In the current study, we investigate psychological drivers of snacking behaviour, including SEP, stress, and the reasons people snack as focal points.

SEP may have a profound and multi-directional effect on snacking behaviour, and on the associations between stress and other emotional reasons people have for snacking. Lower SEP is associated with lower general health and wellbeing [4, 12–14] and a higher BMI [15, 16]. More specifically, low SEP is related to poorer diets from adolescence [17] to adulthood [5], with trends showing an increase rather than a decrease of this disparity over the past decades [6]. When it comes to snacking, some studies suggest that snacking frequency may in fact be higher in high SEP or high income groups [18], but lower SEP has also been associated to more energy dense and less nutrition rich snack choices [19].

The relationship between stress and (unhealthy) eating has also been studied extensively in previous research. In general, whereas short-term, acute threat or stress diminishes appetite [20, 21], long-term, or chronic stress is linked to overeating [22–24]. Increased cortisol levels from long-lasting stress can cloud people’s physical caloric need and lead to an increased preference for highly palatable foods [25, 26]. As snack foods are typically placed in this ‘highly palatable foods’ category, stress increases snacking behaviour [10, 11]. Combining this notion with the observation that our surroundings are increasingly obesogenic [27] and the inevitable reality that life can be stressful [28] underlines the importance of taking stress into account when investigating snacking behaviour. More discrete negative emotions may also play a role. Emotions may not necessarily (or exclusively) have a direct effect, but adaptive and maladaptive emotion regulation strategies that feed into (emotional) eating behaviour may be important (e.g., [29–31]). This implies that focusing on coping with negative emotional states such as sadness, anger, or worrying as a ‘reason to snack’ may further our understanding of why people snack. Taken together, both stress and coping with negative emotions are interesting focal points when studying unhealthy snacking behaviour.

In addition to SEP and stress being considered direct predictors of snacking behaviour, a moderating effect of SEP on associations between stress and snacking behaviour is also plausible. Low SEP is accompanied by constraints and limitations, which is costly in terms of mental and physical resources [4, 32]. This can result in a larger impact of additional stressors on everyday behaviours, including lifestyle behaviours such as unhealthy snacking. As such, one would expect that snacking as a result of stress may be particularly present for those already struggling with the obstacles of a low SEP.

Snacking behaviour may thus be affected by several factors, including psychological rather than biologically driven reasons [33, 34], but insight into their mutual associations and potential other contributing factors is lacking. It is therefore important to identify who is prone to suffering most from stress-related snacking and its unhealthy consequences.

## Current study

To further our understanding of the intricacies of psychological drivers for snacking behaviour, we set up a cross-sectional survey study. First, to test the notion that people with a low SEP would be more susceptible to stress-related reasons for snacking, we tested whether low SEP would lead to more self-reported negative-affect related reasons for snacking under higher levels of stress (a moderation effect)^1^. To get a comprehensive picture of why people snack, we also explored how stress and SEP are associated with other known reasons to snack (i.e., to reward oneself, because of a special occasion, to replenish energy, because of social pressure, or because of the opportunity to do so; [35]). In addition, we focused on how SEP and snacking *behaviour* (‘how often people snack unhealthily?’) were related, including an investigation of stress and the attributions people make about why they snack as underlying processes. Based on the robust literature on SEP and stress and health behaviours, we hypothesized that a lower SEP would be associated with increased snacking frequency, and that stress would mediate this association.

## Methods

### Participants and study design

Participants were recruited via an online panel of a Dutch panel agency (Flycatcher.eu). The authors had full access to the data used in the study. All methods were carried out in accordance with the declaration of Helsinki. Members of the Flycatcher Panel registered voluntarily and gave explicit informed consent to be included in the panel and the current study. Ethical approval for this study was obtained from the ethical review board at Utrecht University. A nationwide sample living across the Netherlands was approached by the panel agency. The recruitment target group consisted of Dutch people aged 18 to 70 years old who had been screened to have no restriction on their eating and snacking behaviour. From 1799 panel members that were approached, 642 participants did not fill in the survey, and 148 participants started, but were excluded based on screening questions regarding their diet. In the end, 1009 participants completed the survey and were included in the data analysis. A post hoc power analysis showed that this would provide us with 0.94 statistical power to detect small (correlation) effects with an alpha of 0.05. Education level was distributed such that 19.7% of the participants had a low level of education (no education, only primary education, or pre-vocational secondary education), 33.2% of the participants had a middle level of education (vocational education, general secondary education, pre-university secondary education, or propaedeutic university or college education), and 47.1% of the participants had a high level of education (a bachelor, master, or higher college or university degree). Income was distributed such that 48.3% earned below a modal income, 15.7% earned a modal income, and 36.1% earned an income exceeding the modal income. Participants were categorized as having a low socio-economic position (SEP) when they earned below a modal income and had a low or middle level of education, or earned a modal income combined with a low level of education. Participants were categorized as having a high SEP when they earned more than the modal income and had a middle or high level of education, or earned a modal income and had a high level of education. This resulted in 58.1% of the participants having a low SEP and 41.9% having a high SEP. BMI was 25.83 on average (*SD* = 4.74; range 15.94–48.32).

### Measures^2^

*Stress.* Life stress was assessed with eight items [36] (e.g., ‘I have to overcome a lot of difficulties’) that participants rated on 5-point Likert scales ranging from 1 (not at all applicable to me) to 5 (very much applicable to me). This scale was reliable with a Cronbach’s Alpha of 0.92, and a mean score indicated amount of stress. In addition, three items on 7-point Likert scales assessed to what extent participants experienced worry due to the COVID-19 pandemic, as data was collected in 2020. Worry in general, and COVID-19-related worry specifically, is a known strong predictor of stress [37–39]. Items were: ‘Because of corona, I worry more’, ‘Because of corona, I worry more about my own health’, and ‘Because of corona, I worry more about the world’. This scale was reliable with a Cronbach’s Alpha of 0.85 and a mean score indicated COVID-19-related worry.

*Reasons for snacking.* Reasons for snacking were assessed with the Reasons for Snacking questionnaire [35]. This scale consists of 22 items, divided into six subscales, one of them being ‘coping with negative emotions’. The other five subscales were ‘opportunity induced eating’, ‘to enjoy a special occasion’, ‘to reward oneself’, ‘because of social pressure’, and ‘to gain energy’. For each item, participants indicated how often they snacked for a specific reason, on a 1 (never) to 7 (always) Likert scale. All subscales displayed good reliability (Cronbach’s Alpha’s > 0.71).

*Snacking behaviour.* To assess snacking behaviour, a Food Frequency Questionnaire was used, a valid and easy-to-access tool to assess frequency of food intake [40, 41]. Participants indicated on how many days over the past month they ate small sweet snacks, large sweet snacks, small savory snacks, and large savory snacks on 9 point scales (1 – none, 2–1–3 days a month, 3–1 day a week, 4–2 days a week, 5–3 days a week, 6–4 days a week, 7–5 days a week, 8–6 days a week, 9 – every day). A mean score for ‘Frequency of snacking’ indicated how many days per week/month on average participants snacked during the past month. In addition, a question was asked to gauge the impact of the COVID-19 pandemic that was ongoing during the data analyses: ‘Do you consume more, less, or the same amount of snacks because of the COVID-19 situation?’.

### Procedure

The selected panel members were contacted by email to participate in the study. Clicking on a personal hyperlink in the email invitation led to the questionnaire. Respondents first answered a verification question to prevent any housemates or others to complete the survey. The survey was only accessible to panel members who received an invitation. Participants first filled out the questionnaires assessing life stress, reasons for snacking, and snacking behaviour. Finally, participants answered demographic questions on age, gender, education level and income and were thanked and paid through panel bonus points.

## Results

### Descriptives

The sample consisted of 47.7% women and 52.3% men. Participants’ mean age was 45.85 years (*SD* = 14.40; range 18–70 years). Table 1 displays means and standard deviations of the variables assessed in the study. Life stress was low to moderate on average with a mean of 2.33 (on a scale of 1–5), and COVID-19 related worry had a moderate mean of 3.73 (on a scale of 1–7), so both life stress and COVID-19 related worry were included as factors in the analyses. Participants indicated to consume snacks on 1–2 days per week on average. To the question whether participants consumed more or less snacks because of the COVID-19 situation, 19.6% indicated to consume more snacks, 72.9% indicated to eat a similar amount of snacks, and 7.4% of participants indicated to consume less snacks.

**Table 1:** Means and standard deviations of variables of interest

	M	SD
Snacking because of.		
…a special occasion	4.09	1.26
… the opportunity	4.06	1.19
…energy	6.76	1.43
…a reward	3.17	1.40
…social pressure	2.58	1.13
…negative emotions	2.68	1.43
Life stress	2.33	0.80
COVID-19 related worry	3.73	1.42
Frequency of snacking	3.72	1.44

### SEP, stress, and reasons to snack

As a first step, correlations were calculated between SEP, life stress, COVID-19-related worry, reasons for snacking, and frequency of snacking. A full correlation table can be found in the supplementary materials. Life stress and COVID-19-related worry were positively correlated (*r* = .29, *p* < .001). SEP was negatively related to life stress (*r* = − .21, *p* < .001) and COVID-19-related worry (*r* = − .19, *p* < .001). SEP was not related to snacking because of negative emotions, or social pressure, but positively related to most reasons to snack: because of a special occasion (*r* = .20, *p* < .001), because of an opportunity (*r* = .15, *p* < .001), to replenish energy (*r* = .11, *p* < .001), and to reward oneself (*r* = .13, *p* < .001). SEP was further positively related to frequency of snacking (*r* = .12, *p* < .001).

Table 2 displays regression coefficients and t-values for regression analyses conducted to test the hypothesis on stress and snacking to cope with negative emotions. When both included in a linear regression model, life stress significantly and positively predicted the tendency to snack to cope with negative emotions. COVID-19 related worry was a marginally significant positive predictor. These results are in line with our hypotheses. Further regression analyses into other reasons for snacking, including life stress and COVID-19 related worry as predictors, revealed that the association is not unique to negative coping with emotions; stress significantly predicted most of the included reasons to snack.

**Table 2:** *Regression coefficients, t-values, and confidence intervals of analyses testing predictive value of life stress and COVID-19 related worry on reasons for snacking*

	Life stress				COVID-19 related worry			
	*β*	*t*	95% CI	*p*	*β*	*t*	95% CI	*p*
Snacking because of.								
…negative emotions	0.36**	11.93	0.54;0.75	< 0.001	0.06^	1.9	− 0.00;0.12	0.058
…opportunity	0.16**	4.82	0.14;0.33	< 0.001	− 0.06^	-1.75	− 0.10;0.01	0.080
…energy	0.15**	4.74	0.16;0.39	< 0.001	0.07*	2.15	0.01;0.13	0.032
…a reward	0.16**	5.01	0.17;0.39	< 0.001	*ns*	*ns*	*ns*	0.226
…social pressure	0.10*	2.94	0.04;0.22	0.003	0.15**	4.52	0.07;0.17	< 0.001
…special occasion	*ns*	*ns*	*ns*	0.689	0.08*	2.92	0.01;0.12	0.022

Note. Coefficients denoted with * are significant with p < .05, coefficients denoted with ** are significant with p < .001, coefficients denoted with ^ are marginally significant with p < .10

To test whether the relationship between life stress and unhealthy snacking to cope with negative emotions was moderated by SEP, the PROCESS macro by Hayes (2017) was used. A moderation model (centred variables, 95% confidence intervals) with life stress as the predictor, tendency to snack because of negative emotions as the outcome variable, and SEP as a moderator, was conducted using the Hayes PROCESS macro [42]. The full model was significant, *R*^2^ = 0.16, *F*(3,1005) = 62.57, *p* < .001, as was the direct effect of life stress on the tendency to snack because of negative affect, *b* = 0.73, *t* = 13.70, *p* < .001, 95%CI [0.63;0.83]. The interaction term life stress x SEP (*b* = 0.26, *t* = 2.29, *p =* .02, 95%CI [0.04;0.47]) significantly predicted coping with negative emotions as a reason to snack. Conditional effects show that the association between life stress and unhealthy snacking to cope with negative emotions is stronger for participants with a high SEP (effect estimated at 0.88, *t* = 9.74, *p* < .001, 95%CI [0.70;1.06] than for participants with a low SEP (effect estimated at 0.63, *t* = 9.63, *p* < .001, 95%CI [0.50;0.75], although both effects were significant. This direction of the moderated effect was in contrast with our original predictions. When running similar moderation analyses with the other reasons to snack, no significant interaction effects with SEP were found.

### SEP and snacking behaviour

To move beyond reasons for snacking and test whether lower SEP would predict more unhealthy snacking behaviour, and whether stress serves as an underlying process in this association, we conducted a mediation analysis using the Hayes (2017) PROCESS macro, to test whether higher stress in individuals with a lower SEP would partially explain any correlation between SEP and snacking frequency. This analysis showed a significant direct and positive effect of SEP on snacking frequency estimated at 0.43, *t* = 4.68, *p* < .001, 95%CI [0.25;0.61]. SEP was a negative predictor of life stress, meaning that a lower SEP predicted more life stress, *R*^2^ = 0.05, *F*(1,1007) = 48.39, *p* < .001, *b = −* 0.35, *t* = -6.96, 95%CI [-0.44;-0.25]. Life stress in turn positively predicted unhealthy snacking frequency *R*^2^ = 0.04, *F*(2,1006) = 18.22, *p* < .001, *b =* 0.27, *t* = 4.72, 95%CI [0.16;0.38], and (partially) explained the association between SEP and snacking frequency in this mediation model, with a significant indirect effect estimated at − 0.09, 95%CI [-0.15;-0.05]. Notably, while SEP *negatively* predicted stress, and life stress *positively* predicted unhealthy snacking frequency (implying that lower SEP is associated with more stress, and more snacking), in this model as well as in simple correlation analyses, SEP in itself is *positively* associated with snacking frequency (implying that higher SEP is associated with a higher unhealthy snacking frequency). Similar statistically significant patterns emerged for COVID-19 related worry as a mediator: a significant direct and positive effect of SEP on snacking frequency was estimated at 0.39, *t* = 4.21, *p* < .001, 95%CI [0.21;0.57] in this analysis. SEP was a negative predictor of COVID-10 related worry, suggesting that a lower SEP predicted more worry, *R*^2^ = 0.04, *F*(1,1007) = 38.79, *p* < .001, *b = −* 0.55, *t* = -6.23, 95%CI [-0.73;-0.38]. COVID-19 related worry in turn positively predicted unhealthy snacking frequency *R*^2^ = 0.02, *F*(2,1006) = 10.95, *p* < .001, *b =* 0.09, *t* = 2.82, 95%CI [0.03;0.15], and (partially) explained the association between SEP and snacking frequency in this mediation model, with a significant indirect effect estimated at − 0.05, 95%CI [-0.09;-0.01].

**Fig. 1:**
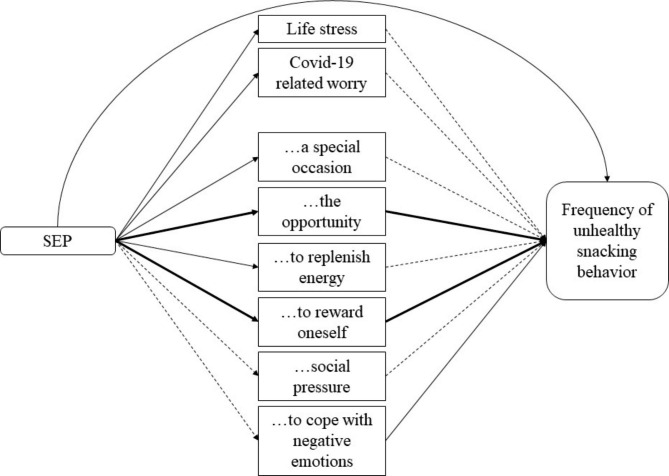
Parallel mediation model to explain the association between SEP and frequency of unhealthy snacking behaviour. Solid arrows are significant associations, dashed arrows are non-significant pathways, and the bold arrows show the significant indirect mediation effects

Finally, to get a comprehensive picture of the pattern of results with regards to SEP and snacking behaviour, we conducted an exploratory parallel mediation analyses using Hayes (2017) PROCESS macro. In these analyses, SEP was included as a predictor, and several mediators were included to predict frequency of snacking: Life stress, COVID-19 related worry, and the different reasons for snacking. This allowed for more insight into the underlying processes in the relationship between SEP and unhealthy snacking behaviour. The tested model is depicted in Fig. 1. The direct effect of SEP on snacking frequency was significant and estimated at 0.17, *t* = 1.99, *p* = .046, 95%CI [0.00;0.35]. as was the total indirect total effect, which was estimated at 0.16, 95%CI [0.06;0.26]. Table 3 provides an overview of the coefficients and confidence intervals of these mediation analyses.

**Table 3:** *Regression coefficients and confidence intervals of indirect effects from the parallel mediation analysis on SEP (coded 0 for low and 1 for high SEP), frequency of unhealthy snacking behaviour, and life stress, COVID-19 related worry, and snacking because of a special occasion, the opportunity, to replenish energy, to reward oneself, because of social pressure, or to cope with negative emotions as mediators*

	Indirect effect	CI 95%
Life stress	0.00	− 0.04; 0.04
COVID-19 related worry	− 0.02	− 0.06; 0.02
Snacking because of.		
…a special occasion	0.02	− 0.02; 0.07
… the opportunity	0.09*	0.04; 0.14
…energy	0.02	− 0.00; 0.04
…a reward	0.05*	0.02; 0.10
…social pressure	− 0.00	− 0.01; 0.01
…negative emotions	0.00	− 0.03; 0.04

Note. Coefficients denoted with * are significant with a confidence interval not containing zero

Results demonstrate that, taken into account parallel mediators, there was no longer a significant mediation between SEP and snacking frequency by life stress or COVID-19 related worry. However, two mediators still played a significant role: snacking because of the opportunity to do so, and snacking to reward oneself. These were both positively predicted by SEP, and positively predicted snacking frequency.

## Discussion

Why and how we snack has been a topic of study due to the increase in snacking behaviour over the past decades, and the adverse health effects of this increase. Focusing on intra-individual factors, most snacking behaviour takes place because of psychological (and not biological) reasons, and may therefore be quite susceptible to variables such as SEP, stress, and negative emotions. The current study findings show that people reporting elevated stress levels (COVID-19 related worry, but also general life stress) attributed their snacking behaviour more to coping with negative emotions. However, with increasing stress, people also attribute their snacking behaviour more to other reasons such as replenishing energy, rewarding oneself, celebrating a special occasion, because of an opportunity that emerges, and because of social pressure. Interestingly, from all six reasons for snacking, snacking to cope with negative emotions was one of the two least frequently indicated reasons overall, implying that negative emotions may play a smaller role than is often portrayed. In general, people seem to find more reasons to snack when they are feeling stressed, which is in line with previous research showing that stress leads to (over)eating [23].

Moreover, we set out to study how SEP may interact with stress levels and affect snacking reasons and snacking behaviour. The association between stress and unhealthy snacking to cope with negative emotions was more pronounced for people with high compared to low SEP. This contrasted with the idea that people with low SEP may suffer from a double burden of pre-existing as well as current heightened stress levels and would therefore perceive coping with negative emotions as a reason to snack. However, it is in line with previous research showing that highly educated groups were more likely to eat unhealthily due to the COVID-19 lockdown [43] as well as the positive correlation between SEP and snacking behaviour from the current study.

Furthermore, we investigated whether SEP was associated with snacking behaviour, moving beyond snacking reasons, and whether stress would underlie this association. Findings revealed interesting, yet somewhat puzzling mediation patterns. In general, SEP is positively associated with unhealthy snacking frequency; people with higher SEP seem to snack more often, which is in contrast with our hypothesis that lower SEP would be associated with more snacking behaviour. These results are in line with positive correlations between SEP and several self-reported attributions or reasons for snacking: because of special occasions, because of the opportunity to do so, to reward oneself, and to replenish energy. Thus, higher SEP was associated with more reasons to snack, and a higher snacking frequency.

However, opposite patterns emerged when stress was factored in. When stress and worry were included as a mediator, *lower* SEP was associated with higher frequency of snacking, partially because of higher stress levels. This was in line with our hypothesis. To add to the complexity of this pattern, a model including stress and reasons for snacking as parallel mediators resulted in two remaining significant mediators, namely snacking because of the opportunity to do so, and snacking to reward oneself. These two reasons statistically explained the positive relationship between SEP and snacking behaviour. Stress was no longer a significant mediator when other reasons for snacking were included, and SEP remained positively associated with snacking frequency, which did not support our hypotheses.

Taken together, results of the study reveal that there is a multitude of factors associated with snacking behaviour, and that their mutual relationships are complex and nuanced. For example, whereas in our study, low SEP was not a risk factor for increased snacking frequency (in fact, higher SEP was associated with more snacking), low SEP was associated with higher levels of stress and worry, which in turn has the potential to contribute to an increase in snacking behaviour. However, when included in a more comprehensive model including more explanatory variables, this association was too weak to be statistically significant.

The results thus showed that higher SEP was associated with more snacking, and that the most pronounced explanatory variables in this association were two reasons to snack: because of the opportunity to do so, and to reward oneself. Many previous studies have pointed out the association between low SEP and an unhealthy eating pattern (e.g., [15, 16]), and in that light, the current results may seem counterintuitive. However, prior research has indicated that under certain circumstances, like the COVID-19 pandemic that was ongoing at the time data was being collected, low income groups may be burdened more by financial uncertainty and thus food-insecurity than high income groups, and that this may lead to decreases in frequency of meals [44, 45]. Similarly, high rather than low education level was associated with increased snack purchases under stressful (in this case, the COVID-19 pandemic) circumstances [43]. SEP comprises facets of income and education level, and therefore results from the current study align with what these previous studies have shown.

Being in a high socio-economic position allows people to allocate more, and potentially other resources compared to being in a low socio-economic position. This may primarily be the case for material resources such as income/money, groceries, and other food purchases. Being able to purchase more snacks, would logically be related to more consumption of snacks. This association is direct, but would also have indirect consequences. When asking people ‘how often do you snack because of….’ followed by a list of several reasons one could have to have a snack, chances are one will indicate having more reasons to snack, because of the actual snacks being more available to them. This may be reflected in ‘because of the opportunity to do so’ being one of the more prominent reasons people with high SEP tend to report as a reason to snack. Moreover, beyond material resources, a higher SEP may also be accompanied by a larger and/or different array of coping mechanisms available to deal with situational demands as well as factors like stress [46, 47]. This may also allow for instrumentally using snacking behaviour, for example to reward oneself, as also indicated by our results. So, whereas low SEP may be associated with elevated stress levels, this does not necessarily need to translate into a maladaptive eating pattern. Contrarily, those with higher SEP should keep an eye out for extensive use of ‘reasons to snack’, which may result in justification of unhealthy or otherwise undesirable behaviours [48].

Several strengths and limitations of the current study must be taken into account. A first strength lies in the inclusion of several potential explanatory variables in the survey, which allowed for getting a relatively comprehensive view of underlying mechanisms, rather than zooming in on one specific variable. Furthermore, we were able to include a large, diverse sample with a substantial amount of people with a low SEP, which is often not the case in survey studies. As for limitations, the most evident reason for caution when interpreting the results lies in the cross-sectional nature of the data: no claims with regards to causality can be made based on the design and data. Moreover, caution is warranted when interpreting the results due to most effects being relatively small, and multiple testing having taken place. However, it is also worth noting that small effects are common in the field of psychology and meaningful in their own right [49]. Furthermore, although self-report is a valuable way of measuring psychological variables, an additional more objective measure of snacking behaviour (e.g., a snacking diary) would be a useful addition to the design. In addition, although most measures were based on existing measures for stress, reasons for snacking, and snacking frequency, including other validated measures would improve future studies. Moreover, although the sample was large and varied, there may still have been selection bias as potentially participants who were interested in the topic decided to accept the invitation for the survey. Finally, data collection took place during the COVID-19 pandemic, and although the majority of participants indicated not to have changed their snacking behaviour because of this, multiple studies have shown the effects of these circumstances on eating behaviour [43, 50–53], so a replication post-pandemic would be a valuable addition to this research line.

## Conclusion

In conclusion, snacking forms a significant share of people’s dietary patterns, and its rising prevalence and mal-nutritious characteristics call for more understanding of what variables predict how much people snack. Although stress is often mentioned as a predictor of snacking behaviour, this study demonstrated that many other reasons to snack seem to trump stress when it comes to self-reported attributions about snacking behaviours. Furthermore, results from this study show us that SEP is associated with behaviour in many ways, and that when it comes to snacking behaviour, a higher SEP allows for more reasons to snack, and ultimately more frequent snacking.

## Electronic supplementary material

Below is the link to the electronic supplementary material.

Supplementary Material 1

## Data Availability

The materials and data that support the findings of this study are available from the corresponding author, MG, upon reasonable request.

## Footnotes

1 The study and moderation hypothesis were pre-registered on AsPredicted.org: https://aspredicted.org/8fm7f.pdf. Data and syntax: https://osf.io/vmg76/?view_only=89f9054a71ce4cc3aee43ee9cdcc3a3f.

2 Additionally, questions assessing neighborhood social norms, loneliness, sense of mastery, boredom, healthy eating importance, and snack portion size were included in the survey. These measures were deemed beyond the scope of the current study and are therefore not elaborated upon
