# Supplementary material for: Snacking for a reason: detangling effects of socio-economic position and stress on snacking behaviour
Source: BMC Public Health. 2022 Nov 2;22:2009. doi: 10.1186/s12889-022-14384-2 (PMC9628631; doi:10.1186/s12889-022-14384-2)
Supplement: Supplementary file 1 — Supplementary Material 1 [file 12889_2022_14384_MOESM1_ESM.docx]

**Appendix A**

Table

*Correlations between SEP, stress, reasons for snacking, and frequency of snacking behaviour (N = 1009)*

|  | SEP (1) | Life stress (2) | COVID-19 worry (3) | …a special occasion (4) | …the opportunity (5) | …for energy (6) | …as a reward (7) | … social pressure (8) | … negative emotions (9) | Frequency of snacking  (10) |
| --- | --- | --- | --- | --- | --- | --- | --- | --- | --- | --- |
| (1) |  | -.21** | -.19** | .20** | .15** | .11** | .13** | .01 | .01 | .12** |
| (2) |  |  | .29** | .04 | .14** | .17** | .17** | .14** | .38** | .12** |
| (3) |  |  |  | .08* | -.01 | .11** | .09** | .17** | .16** | .06* |
| (4) |  |  |  |  | .53** | .31** | .47** | .49** | .27** | .28** |
| (5) |  |  |  |  |  | .39** | .53** | .38** | .41** | .39** |
| (6) |  |  |  |  |  |  | .36** | .26** | .22** | .24** |
| (7) |  |  |  |  |  |  |  | .48** | .56** | .38** |
| (8) |  |  |  |  |  |  |  |  | .44** | .23** |
| (9) |  |  |  |  |  |  |  |  |  | .35** |

Note. Correlations denoted with * are significant with p<.05, coefficients denoted with ** are significant with p<.001.
